# Identification of an Exceptionally Long Intron in the *HAC1* Gene of *Candida parapsilosis*

**DOI:** 10.1128/mSphere.00532-18

**Published:** 2018-11-07

**Authors:** Elise Iracane, Paul D. Donovan, Mihaela Ola, Geraldine Butler, Linda M. Holland

**Affiliations:** a School of Biomedical and Biomolecular Science and UCD Conway Institute of Biomolecular and Biomedical Research, Conway Institute, University College Dublin, Dublin, Ireland; Carnegie Mellon University

**Keywords:** *Candida parapsilosis*, Hac1, introns, unfolded protein response

## Abstract

The unfolded protein response (UPR) in the endoplasmic reticulum (ER) is well conserved in eukaryotes from metazoa to yeast. The transcription factor *HAC1* is a major regulator of the UPR in many eukaryotes. Deleting *HAC1* in the yeast Candida parapsilosis rendered cells more sensitive to DTT, a known inducer of the UPR. The deletion strain was also sensitive to Congo red, calcofluor white, and the antifungal drug ketoconazole, indicating that *HAC1* has a role in cell wall maintenance. Transcriptomic analysis revealed that treatment of the wild type with DTT resulted in the increased expression of 368 genes. Comparison with mutant cells treated with DTT reveals that expression of 137 of these genes requires *HAC1*. Enriched GO term analysis includes response to ER stress, cell wall biogenesis and glycosylation. Orthologs of many of these are associated with UPR in Saccharomyces cerevisiae and Candida albicans. Unconventional splicing of an intron from *HAC1* mRNA is required to produce a functional transcription factor. The spliced intron varies in length from 19 bases in C. albicans to 379 bases in Candida glabrata, but has not been previously identified in Candida parapsilosis and related species. We used RNA-seq data and *in silico* analysis to identify the *HAC1* intron in 12 species in the CTG-Ser1 clade. We show that the intron has undergone major contractions and expansions in this clade, reaching up to 848 bases. Exposure to DTT induced splicing of the long intron in C. parapsilosis *HAC1*, inducing the UPR.

**IMPORTANCE** The unfolded protein response (UPR) responds to the build-up of misfolded proteins in the endoplasmic reticulum. The UPR has wide-ranging functions from fungal pathogenesis to applications in biotechnology. The UPR is regulated through the splicing of an unconventional intron in the *HAC1* gene. This intron has been described in many fungal species and is of variable length. Until now it was believed that some members of the CTG-Ser1 clade such as *C. parapsilosis* did not contain an intron in *HAC1*, suggesting that the UPR was regulated in a different manner. Here we demonstrate that *HAC1* plays an important role in regulating the UPR in C. parapsilosis. We also identified an unusually long intron (626 bp) in C. parapsilosis *HAC1*. Further analysis showed that *HAC1* orthologs in several species in the CTG-Ser1 clade contain long introns.

## Abstract (precis)

The unfolded protein response (UPR) responds to the build-up of misfolded proteins in the endoplasmic reticulum. The UPR has wide-ranging functions from fungal pathogenesis to applications in biotechnology. The UPR is regulated through the splicing of an unconventional intron in the *HAC1* gene. This intron has been described in many fungal species and is of variable length. Until now it was believed that some members of the CTG-Ser1 clade such as *C. parapsilosis* did not contain an intron in *HAC1*, suggesting that the UPR was regulated in a different manner. Here we demonstrate that *HAC1* plays an important role in regulating the UPR in C. parapsilosis. We also identified an unusually long intron (626 bp) in C. parapsilosis *HAC1*. Further analysis showed that *HAC1* orthologs in several species in the CTG-Ser1 clade contain long introns.

## INTRODUCTION

The unfolded protein response (UPR) is activated in response to the build-up of misfolded proteins in the endoplasmic reticulum (ER). Expression of genes required to deal with the ER stress is induced during the UPR (1). The UPR response in fungi was first characterized in the model yeast Saccharomyces cerevisiae (2). The UPR is triggered by a transmembrane sensor, Ire1 (inositol requiring enzyme 1), which senses the accumulation of misfolded proteins. Ire1 is an endonuclease that cleaves and removes an atypical intron from *HAC1* mRNA (3). This facilitates translation of the bZIP transcription factor Hac1, which subsequently regulates the expression of genes required for the UPR (1). Hac1 binds to the UPR elements (UPRE) present in the promoter regions of ER-chaperone genes such as *KAR2/*BiP and induces gene transcription (4). The response to unfolded proteins is evolutionarily conserved and plays a central role in the ER stress response in eukaryotes (5).

The UPR is important for fungal pathogenesis (5, 6). In Candida albicans Hac1 is required for hyphal formation, which is an important aspect of virulence for this pathogen (7). The UPR is also important for antifungal activity, as UPR-impaired mutants in C. albicans are more sensitive to chemicals such as carvacrol (8). The UPR is also required for the virulence and antifungal resistance of Aspergillus fumigatus (9). In Candida glabrata, Ire1 was found to be required for ER stress but acts independently of Hac1 (10).

The activity of Hac1 has been exploited for biotechnology applications. Pichia pastoris (Komagataella phaffii) is a widely used system for protein production, and studies have shown that high-level expression of heterologous protein can induce the UPR (11). This can be overcome by overexpressing the spliced form of *HAC1*, increasing the production of heterologous proteins (12, 13). The same method was used in Aspergillus niger var. *awamori* and in Trichoderma reesei to increase the yield of secreted heterologous protein (14, 15). Moreover, in T. reesei protein secretion is regulated not only by the UPR but by another stress response system named REpression under Secretion Stress (RESS) (16).

Ire1-mediated splicing of the *HAC1* intron has been described in many fungi, including T. reesei, A. nidulans, C. albicans, Yarrowia lipolytica and P. pastoris (4, 7, 17, 18). The overall structure of the intron is well conserved. Common features include two short hairpins at the exon/intron boundaries with the splice sites located within these regions (19). However, the length of the intron varies from 19 nucleotides in C. albicans to 379 nucleotides in C. glabrata (19).

C. albicans belongs to the CTG-Ser1 clade (species where CTG is translated as serine rather than leucine) (20, 21). Hooks and Griffiths-Jones (19) showed that in some species in the CTG clade (including C. albicans, Candida tropicalis, and Candida dubliniensis) the *HAC1* intron is very short (between 19 bp and 22 bp). However, they could not identify the intron in the other CTG-Ser1 species, including C. parapsilosis, Lodderomyces elongisporus, Debaryomyces hansenii, Scheffersomyces stipitis, Clavispora lusitaniae and Meyerozyma guilliermondii, suggesting that these species may use an alternative mechanism to regulate the UPR (19).

Here we describe the role of *HAC1* in the C. parapsilosis UPR. Deletion of *HAC1* renders strains susceptible to ER stress. RNA-seq experiments further confirm a role for C. parapsilosis *HAC1* in the ER stress response. We also show that there is an exceptionally long intron (626 nucleotides) in C. parapsilosis *HAC1*, which is spliced under ER stress growth conditions. *HAC1* genes in other CTG-Ser1 clade species also contain unusually long introns.

## RESULTS AND DISCUSSION

### Functional characterization of C. parapsilosis *HAC1*.

**FIG 1:**
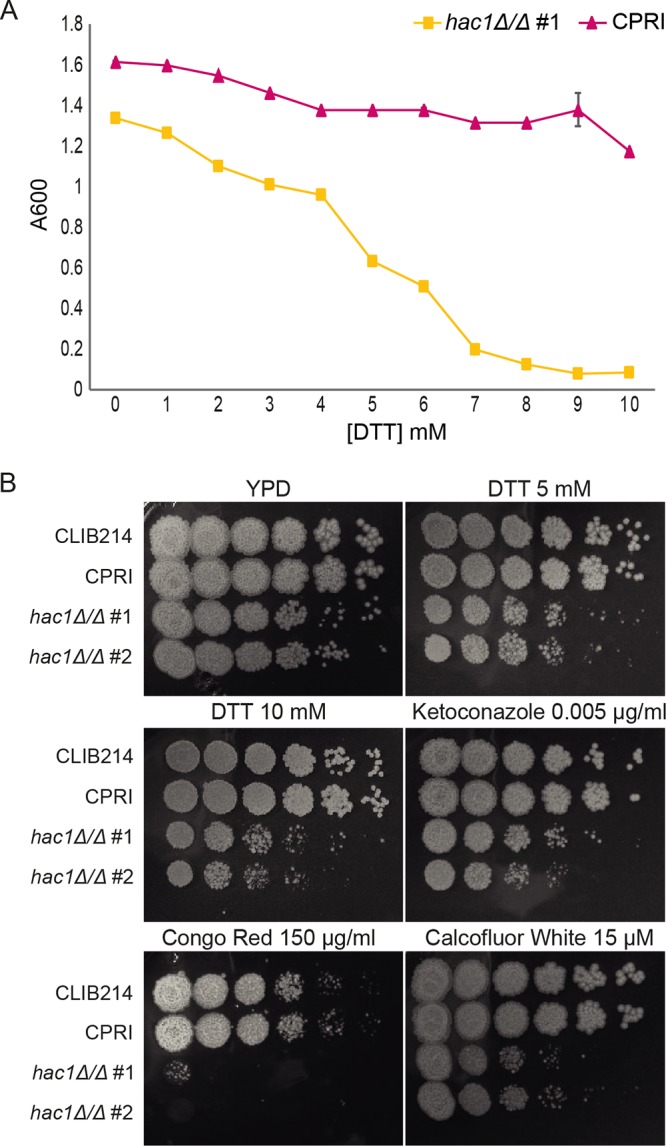
Role of *HAC1* in stress response. (A) Growth at 24 h of the C. parapsilosis CPRI control strain and the *hac1Δ/Δ* #1 mutant incubated with DTT (0 to 10 mM) in liquid YPD at 30°C. (B) Growth of C. parapsilosis CLIB214, CPRI, *hac1Δ/Δ* #1 and *hac1Δ/Δ* #2 on solid YPD or YPD supplemented with DTT (5 mM and 10 mM), ketoconazole (0.005 µg/ml), Congo red (150 µg/ml), and calcofluor white (15 µM). Plates were incubated at 30°C for 48 h.

A putative *HAC1* gene, *CPAR2_103720*, was identified in the C. parapsilosis genome based on sequence similarity to other *HAC1* orthologs (22, 23). Previous studies have shown that *HAC1* has a core role in the UPR (7). Deleting *CPAR2_103720* in C. parapsilosis resulted in increased sensitivity to DTT (a strong reducing agent that induces the UPR by preventing disulfide-bond formation) in comparison to the control strain CPRI (24) (Fig. 1A). Although the *hac1Δ/Δ* mutants displayed a growth defect when grown on YPD agar, growth is significantly more reduced in the presence of DTT (Fig. 1B). C. parapsilosis Hac1 therefore plays an important role in the UPR, similar to other fungal species.

Deleting *HAC1* also increased sensitivity to Congo red (interferes with glucan synthesis and cross-linking [25]), calcofluor white (interferes with glucan synthesis and cross-linking [26]) and the antifungal ketoconazole (Fig. 1B). Similar phenotypes were observed in C. albicans *hac1* deletions (7–9). These results indicate that *HAC1* plays an essential role in *Candida* species in regulating the response to cell wall stress. Maintaining cell wall integrity is essential for normal cell growth, division, hypha formation, and antifungal tolerance (27).

### Transcriptional profiling of the *hac1* deletion.

**FIG 2:**
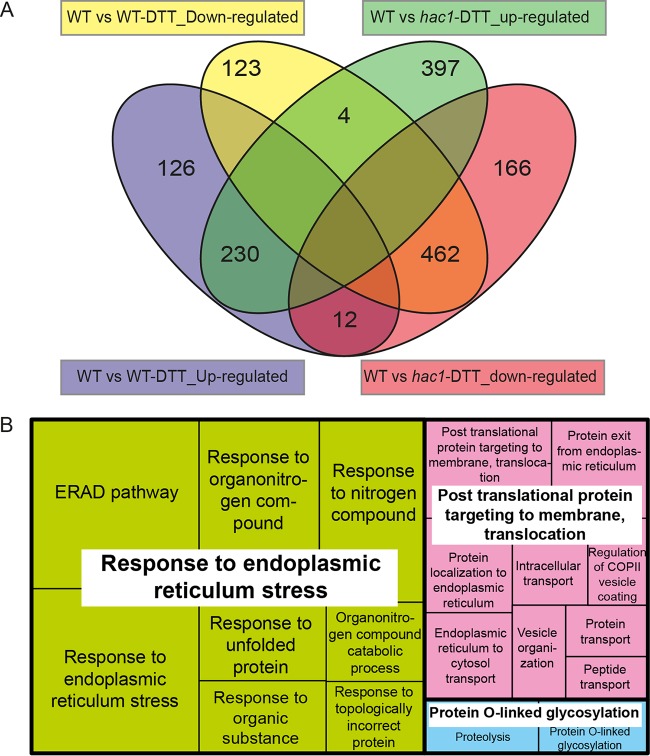
Transcriptomic analysis of UPR induction in CLIB214 and *hac1Δ/Δ* strains. (A) Genes upregulated and downregulated in WT and *hac1Δ/Δ* strains following 1-h exposure to 5 mM DTT. (B) Associated GO terms for biological process of the 138 Hac1-dependent genes. The TreeMap has been generated by REVIGO (48); each tile size is proportional to the absolute log_10_ of the *P* value of each GO ID.

RNA-seq was used to identify the targets of Hac1 in C. parapsilosis under UPR. Exponentially growing C. parapsilosis CLIB214 (wild type) and *hac1Δ/Δ* strains were grown in YPD with and without exposure to DTT for 1 h. Exposing C. parapsilosis CLIB214 to DTT resulted in increased expression of 368 genes and decreased expression of 589 genes (Fig. 2A). Ribosome biogenesis and assembly genes are downregulated, similar to C. albicans and S. cerevisiae (7). Upregulated genes are enriched for categories such as ERAD pathway and response to endoplasmic reticulum stress, which are associated with the UPR response in C. albicans (7). When *HAC1* is deleted, 230 genes remain upregulated when DTT is added. Therefore, expression of these genes is Hac1-independent. We found that expression of 138 genes was no longer induced by DTT when *HAC1* was deleted. From this group of 138 genes, expression of 126 genes (see Table S1 posted at https://figshare.com/s/1f2fc034a73948fe0963) was no longer upregulated in DTT-treated *HAC1* mutant cells, and expression of 12 genes (including *HAC1* [see Table 3]) was reduced (Fig. 2A). GO term analysis of these 12 Hac1-dependent genes identified enrichment of processes related to response to endoplasmic reticulum stress, posttranslational protein targeting to membrane and protein O-linked glycosylation (Fig. 2B). Orthologs of several of these genes are also *hac1*-dependent in other species such as in C. albicans (6 genes [7]), S. cerevisiae (7 genes [1, 28, 29]) and *H. polymorpha* (4 genes [30]). Three are required for virulence in C. albicans: *PMT1* (*CPAR2_704010*), *PMT4* (*CPAR2_104900*) and *SERP1* (*CPAR2_102440*) (31, 32). *CPAR2_602430*, which is regulated by Hac1 in C. parapsilosis but not in C. albicans, is upregulated during infection of THP-1 monocytes by C. parapsilosis (33). Hac1 is therefore likely to be important for pathogenicity in both species.

### Identification of a noncanonical intron in C. parapsilosis *HAC1.*

**FIG 3:**
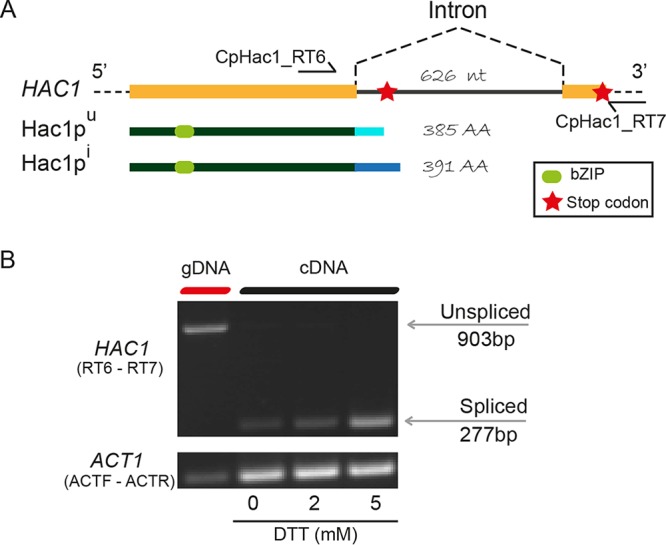
*HAC1* splicing during UPR. (A) Schematic representation of *HAC1*, Hac1p uninduced (Hac1p^u^) and Hac1p induced (Hac1p^i^). Predicted bZIP domains are represented in light green; stop codon is represented by a red star. (B) RT-PCR of C. parapsilosis CLIB214 (WT) cells treated with 0 mM, 2 mM or 5 mM DTT for 1 h. CpHac1_RT6 and CpHac1_RT7 primers (shown in panel A) used to amplify *HAC1*. Actin was amplified using primers ACTF and ACTR (Table 2). A control using genomic DNA is shown in the first lane.

Hooks and Griffiths-Jones (19) carried out a detailed investigation of *HAC1* introns in many fungal species. However, they could find only the hairpin flanking the 5′ splice sites but not the 3′ splice sites of putative introns in *HAC1* in C. parapsilosis and four other species in the CTG-Ser1 clade, and they could not identify any intronic features in C. lusitaniae. We used a splice-aware tool, HISAT2 (34), to map RNA-seq data for C. parapsilosis (35), and identified both splice sites in a putative intron in the last third of the ORF of the gene (Fig. 3A). The intron is unusually long (626 bp), in comparison to that of C. albicans (19 nucleotides) or S. cerevisiae (252 nucleotides) (7).

We next used RT-PCR to determine if splicing of the long intron is regulated by the UPR. Genomic DNA was used as a control to amplify the unspliced PCR product (903 bp) (Fig. 3B). Exposure to DTT induced the removal of the intron (Fig. 3B). There is evidence of spliced and unspliced products when cells are grown in the absence of DTT (Fig. 3B). The very low level of unspliced product in the absence of stress has been observed in other yeast species, including P. pastoris (18). However, when DTT is added, the amount of spliced product is greatly increased, suggesting that splicing of C. parapsilosis *HAC1* is regulated in a similar manner to other fungi (Fig. 3B) (4, 7, 9).

When the intron is not removed, the predicted Hac1p^u^ protein (uninduced form of Hac1) is 385 amino acids long because of a premature stop codon in the intron. Splicing of the intron will generate a Hac1p^i^ protein (induced form) of 391 amino acids (Fig. 3A). Hac1p^i^ is only 6 amino acids longer than Hac1p^u^. However, the C-terminal tail differs by 33 amino acids. The bZIP domain is conserved in both versions of the protein (36) (Fig. 3A). This is the domain that binds to the DNA to activate the target genes (37), suggesting that the presence of the bZIP domain is not enough to activate Hac1p. Cox and Walter (2) showed that in S. cerevisiae the Hac1p^u^ C-terminal tail was extremely unstable, leading to a rapid degradation of the protein.

### Identification of the atypical intron in other CTG-Ser1 clade species.

**FIG 4:**
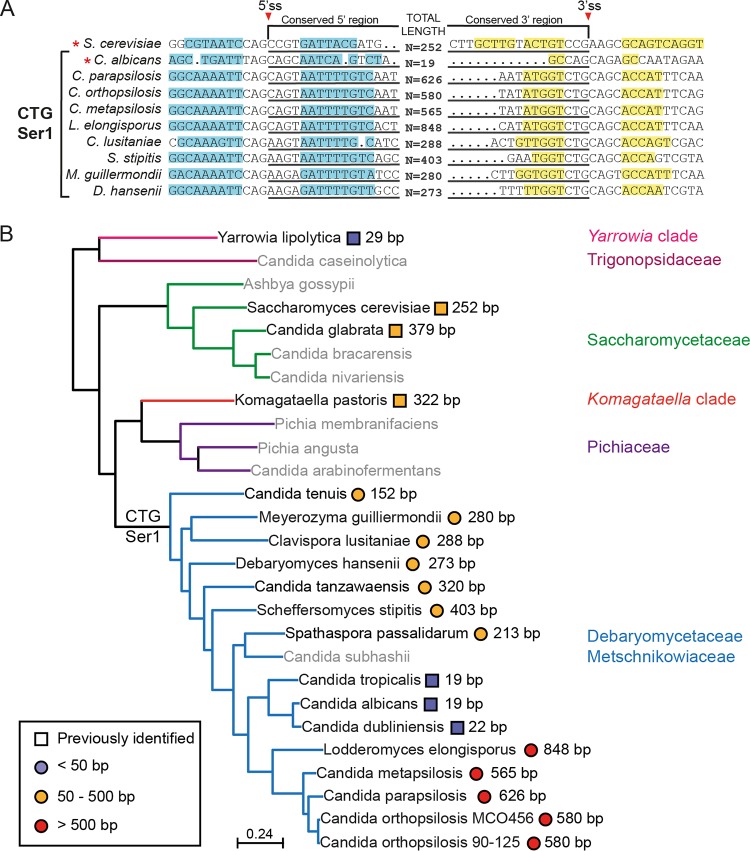
*HAC1* intron across CTG-Ser1 clade. (A) Alignment of *HAC1* from 10 species. The 5′ and 3′ splicing sites are indicated. Red asterisks indicate previously identified introns. The stem-loop complementary regions are highlighted in blue and yellow. (B) Phylogenetic tree represents the *HAC1* intron size through the Saccharomycotina. Species shown in gray were not examined (Candida caseinolytica, Ashbya gossypii, Candida bracarensis, Candida nivariensis, Pichia membranifaciens, Pichia angusta, Candida arabinofermentans, and Candida subhashii). The species where the intron was previously identified are represented by squares, and those with introns newly identified during this study are represented by circles.

We next used RNA-seq data to identify introns in *HAC1* in other species in the CTG-Ser1 clade, including Candida orthopsilosis, Lodderomyces elongisporus (35), Scheffersomyces stipitis (38), and Clavispora lusitaniae (39). Introns were predicted by manual inspection in *HAC1* orthologs in Candida tenuis, Meyerozyma guilliermondii, Debaryomyces hansenii, Candida tanzawaensis, Spathaspora passalidarum, and Candida metapsilosis (Fig. 4). All of the features of the intron are present in all species, including two hairpin loops surrounding the 5′ and 3′ splice sites (Fig. 4A). The newly identified introns vary in length from 152 nucleotides in C. tenuis to almost 850 nucleotides in L. elongisporus. The unexpectedly long intron length and an incorrect annotation of the *HAC1* open reading frame in C. albicans orthologs may explain why these introns were not previously identified (19).

Figure 4B shows the distribution of intron length in *HAC1* orthologs in the Saccharomycotina (tree adapted from Pryszcz et al. [40]). Introns fall into three main groupings: small (<50 nucleotides), medium (50 to 500 nucleotides), and large (>500 nucleotides). Orthologs from related species have introns of similar length. For example, species within the *Saccharomycetaceae* (e.g., S. cerevisiae and C. glabrata) have medium-length introns. It is likely that the ancestor of the Saccharomycotina also had a medium-length intron, because similar lengths are observed in species in the *Komagataella* clade (*K. pastoris*) and some members of the CTG-Ser1 clade (e.g., C. lusitaniae). However, there have been major contractions and expansions in intron size in other CTG-Ser1 clade species. In C. albicans, C. dubliniensis, and C. tropicalis the introns are very short (19 to 22 nucleotides), whereas in C. parapsilosis and related species the introns are very long (up to 840 nucleotides).

### Conclusion.

The UPR is essential for optimizing the response to endoplasmic reticulum stress and the build-up of misfolded proteins, and in many eukaryotes is activated by splicing of a noncanonical intron in *HAC1*. In contrast to a previous report (19), we show that the intron is present in *HAC1* orthologs throughout species in the CTG-Ser1 clade. However, the length of the intron varies substantially within this clade, ranging from 19 nucleotides to 848 nucleotides. The length of the intron does not appear to alter the function or regulation of *HAC1*; orthologs in C. albicans (19 nucleotides) and C. parapsilosis (626 nucleotides) are spliced in a similar manner (7), and regulate expression of ERAD and other stress genes.

## MATERIALS AND METHODS

### Strains, media, and growth.

All strains are listed in Table 1, and all primers are listed in Table 2. Yeast strains were grown in liquid YPD (2% glucose, 2% peptone, 1% yeast extract) supplemented with 1 mM to 10 mM dithiothreitol (DTT; Sigma-Aldrich D0632) where indicated. For phenotype analysis, yeast cells from an overnight culture were washed twice in PBS, diluted to an *A*_600_ of 1 in PBS, and serially diluted 1:5 five times in a 96-well plate. Dilutions were pinned with a 48-pin replicator to YPD agar, supplemented with 5 mM DTT, 10 mM DTT, 0.005 µg/ml ketoconazole, 150 µg/ml Congo red or 15 µM calcofluor white where indicated. Plates were incubated at 30°C for 48 h. *CPAR2_103720* (*HAC1*) was deleted in C. parapsilosis CPL2H1 by replacement of one allele with *HIS1* from C. dubliniensis and the second with *LEU2* from Candida maltosa by homologous recombination as described previously (41). Two *HAC1* deletion strains were generated by deleting the first *HAC1* allele to create a heterozygous mutant. The second allele was deleted in this heterozygous strain and two individual mutants were chosen called C. parapsilosis *hac1Δ/Δ* #1 and *hac1Δ/Δ* #2. CPRI is the control strain with integration of *CdHIS1* and *CmLEU2* at the site of the original *HIS1* alleles. All primers used are listed in Table 2. Upstream and downstream regions were amplified using Q5 High-Fidelity DNA polymerase (New England BioLabs) with, respectively, CpHAC1_KO1 and CpHAC1_KO3 primers, and CpHAC1_KO4 and CpHAC1_KO6 primers. The selectable markers *HIS1* and *LEU2* were amplified using *Ex Taq* polymerase (TaKaRa) with CpHAC1_KO2 and CpHAC1_KO5. The CpHAC1_KO5 primer introduces a unique barcode into the deletion strain (Table 2). The upstream region, one of the selectable markers and the downstream region were fused by PCR using *Ex Taq* Polymerase (TaKaRa) and the resulting disruption cassette was transformed into C. parapsilosis CPL2H1 by chemical transformation as described previously (41). Correct insertion of marker was confirmed by PCR using CpHAC1_5′Check_2 and LEU_Check_1/HIS_Check_1 in the 5′ region and CpHAC1_3′Check and LEU_Check_2/HIS_Check_2 in the 3′ region. Complete open reading frame deletion was confirmed using primers CpHac1_ORF_F and CpHac1_Check_R.

**TABLE 1:** Strains used in the study

Strain	Species	Genotype	Source or reference
CLIB214	C. parapsilosis	Wild type	Type strain
CPRI	C. parapsilosis	*leu2*::*FRT/leu2*::*FRT his1*::*FRT/his1*::*FRT frt*::*CmLEU2/frt*::*CdHIS1*	41
CPL2H1	C. parapsilosis	*leu2*::*FRT/leu2*::*FRT his1*::*FRT/his1*::*FRT*	41
*hac1*Δ#1	C. parapsilosis	*leu2*::*FRT/leu2*::*FRT his1*::*FRT/his1*::*FRT hac1*::*CmLEU2/hac1*::*CdHIS1*	This study
*hac1*Δ#2	C. parapsilosis	*leu2*::*FRT/leu2*::*FRT his1*::*FRT/his1*::*FRT hac1*::*CmLEU2/hac1*::*CdHIS1*	This study

**TABLE 2:** Primers used in the study

Primer	Sequence
RT-PCR	
CpHac1_RT6	TGGGAAACTTTTCACAAAATACG
CpHac1-RT7	TCACACCATAAATCAATCCAACTC
ACTF	GAAGCTTTGTTCCGTCCAGC
ACTR	TGATGGAGCCAAAGCAGTGA
*HAC1* deletion in Candida parapsilosis	
CpHAC1_KO1	ATACCCCCTTTGGATCAATT
CpHAC1_KO3	CACGGCGCGCCTAGCAGCGGGACTAGTATGTGTGGGCTTA
CpHAC1_KO2	CCGCTGCTAGGCGCGCCGTGACCAGTGTGATGGATATCTGC (universal primer)
CpHAC1_KO5	GCAGGGATGCGGCCGCTGAC**GCGCAACCTTCCGGAGTAT**AGCTCGGATCCACTAGTAACG^*a*^
CpHAC1_KO4	GTCAGCGGCCGCATCCCTGCAATAATCAAGTTATTTTTAG
CpHAC1_KO6	CCTCATTCAGTGGGAGTG
CpHAC1_5′Check_2	CGATGAAACGCAGTAGCAAA
CpHAC1_3′Check	TATAACACAAGAAAACAATC
LEU check 1	GAAGTTGGTGACGCGATTGT
LEU check 2	TTCCCCTTCAATGTATGCAA
HIS check 1	AAAATCAATGGGCATTCTCG
HIS check 2	TGGGAAGCAGACATTCAACA
CpHac1_check_R	TTTCCACCTCTTCTTGAACCA
CpHac1_ORF_F	CCACCTAGGAAGAGAGCCAAG

a The barcode present in primer CpHAC1_KO5 is shown in bold and underlined.

### RNA extraction.

C. parapsilosis CLIB214, *hac1Δ/Δ* #1, and *hac1Δ/Δ* #2 were grown overnight in YPD, inoculated in 60 ml YPD to an *A*_600_ of 0.2, and incubated at 30°C with shaking until *A*_600_ of 0.6 was reached. Fifteen milliliters of this culture was then supplemented with H_2_O (control) or 5 mM DTT and incubated for 1 h at 30°C. The cells were collected, resuspended in 200 µl RNAlater, snap-frozen in liquid nitrogen, and stored at −80°C. RNA was extracted using a RiboPure RNA purification kit (yeast) (Ambion; catalog no. AM1926). Quality was assessed by Agilent 2100 Bioanalyzer instrument.

### RT-PCR.

For each sample 2 µg of RNA was treated with 2 U of DNase I (Invitrogen 18068015) in a total volume of 20 µl for 5 min at room temperature, followed by DNase inactivation by adding 1 µl of 25 mM EDTA and incubating at 65°C for 10 min. To synthesize cDNA, 5 µl of DNase-treated RNA was incubated at 70°C for 10 min with oligo(dT) (Promega) at a final concentration of 20 µg/ml. One microliter RNasin (40 U/ml), 4 µl 5× MMLV-RT buffer, 1 µl dNTPs (10 mM), 1 µl MMLV-RT enzyme and 7 µl RNase free H_2_O were added to the cDNA mix and incubated for 1 h at 37°C and then for 2 min at 95°C. The cDNA was then amplified using primers CpHac1_RT6/CpHac1_RT7 (Table 2) to detect splicing of the intron in Hac1 RNA and primers ACTF/ACTR as a control to amplify actin.

### RNA sequencing and analysis.

RNA extracted from 12 samples was sequenced by BGI Global Genomics Services (100 bases, paired-end reads; over 10 million reads per sample). The samples were 3 biological replicates of CLIB214 (WT) incubated for 1 h with 5 mM DTT or with H_2_O, 2 biological replicates of *hac1Δ/Δ* #1 incubated 1 h with 5 mM DTT or with H_2_O, and 1 replicate of *hac1Δ/Δ* #2 incubated 1 h with 5 mM DTT or with H_2_O. Data were analyzed using established bioinformatic protocols (42). Raw paired-end sequenced reads were trimmed using Skewer v0.1.120 (43) and mapped to the Candida parapsilosis CDC317 genome using TopHat v2.0.12 (44). Transcripts were counted using htseq-count from HTSeq v0.6.1 (45), and differentially expressed genes were identified using the Bioconductor package DESeq2 (46). A log_2_FC of >1 or <−1 and an adjusted *P* value of <0.001 were used as cutoff values (Table 3).

**TABLE 3:**
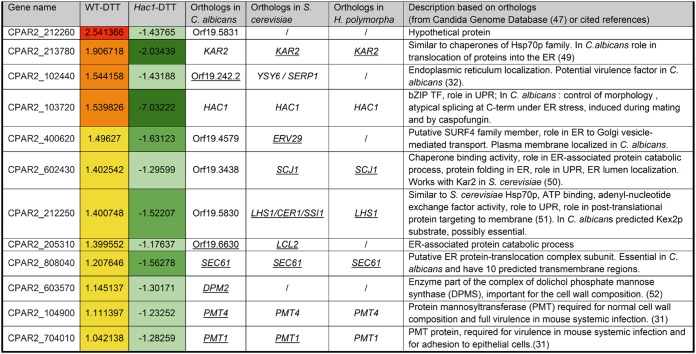
List of 12 genes downregulated in *hac1Δ/Δ* strain involved in UPR^*a*^

a Differential gene expression in fold change in WT cells exposed to DTT (column 2) and in *hac1Δ/Δ* cells exposed to DTT (column 3) compared to WT untreated cells. Color for log fold change: 1> and <1.5, yellow; 1.5> and <2, orange; 2>, red; −1> and <−1.5, light green; −1.5> and <−2, medium green; −2>, dark green. Orthologs found to be Hac1 dependent in *C. albicans*, *S. cerevisiae*, or *H. polymorpha* are underlined.

Gene ontology was found by CGD Gene Ontology Term Finder using default setting (47). REVIGO (48) has been used to generate TreeMap using the default settings (allowed similarity: medium [0.7], semantic similarity measure used: SimRel) in the database with GO term sizes: Saccharomyces cerevisiae.

### Identification of intron in *HAC1* in several species.

HISAT2 (v2.0.4) (34), a splice-aware RNA-seq mapping tool, was used to map RNA-seq data to complete genomes where available, including C. parapsilosis, Candida orthopsilosis and Lodderomyces elongisporus (SRP077251) (35), Pichia (Scheffersomyces) stipitis (SRX135712) (38), and Clavispora lusitaniae (SRX1131478) (39). Intron predictions were manually inspected by comparing against known *HAC1* intron structures from related species. Where no RNA-seq data were available, the *HAC1* intron structure was identified by manual alignment with known *HAC1* genes (Candida tenuis, Pichia [Meyerozyma] guilliermondii, Debaryomyces hansenii, Candida tanzawaensis, Spathaspora passalidarum, and Candida metapsilosis).

### Data availability.

RNA-seq data sets used for intron identification for C. parapsilosis, Candida orthopsilosis and Lodderomyces elongisporus are available using the accession number SRP077251 (35), for Pichia (Scheffersomyces) stipitis at SRX135712 (38), and for Clavispora lusitaniae at SRX1131478 (39). The raw gene expression data for the C. parapsilosis *HAC1* RNA-seq experiment are available at the Gene Expression Omnibus database under accession number GSE120094. Table S1 is available at https://figshare.com/s/1f2fc034a73948fe0963.
